# Commentary: Religious credence is not factual belief

**DOI:** 10.3389/fpsyg.2016.01544

**Published:** 2016-10-13

**Authors:** Konrad Talmont-Kaminski

**Affiliations:** Psychology Faculty, University of Finance and ManagementWarsaw, Poland

**Keywords:** religious credence, religious belief, prosocial belief, prosocial religion, science-religion distinction

Van Leeuwen (2014) claims religious credences are not factual beliefs. He holds that while factual beliefs alone (i) guide behavior in all relevant practical settings, (ii) support inferences between religious credences and (iii) are evidentially vulnerable; religious credences instead (a) have a perceived normative orientation, (b) are open to free elaboration and (c) are vulnerable to special authority. However, it is relatively easy to think of potential counterexamples to that seemingly neat dichotomy, as Van Leeuwen allows. Thus, rituals—such as rain dances—often have purported mundane effects that render beliefs regarding them evidentially vulnerable. The beliefs regarding such rituals must support inferences between factual claims about the details of the ritual to be performed and its efficacy, and certainly do guide ritual behavior in the relevant practical setting. The literature on ritual failure seems to bear out such a more heterogenous view of religious beliefs (Hüsken, 2007).

Yet, it is not easy to judge the significance of such examples. Van Leeuwen is right to point out, after all, that claims normally deemed to be religious may have the traits of factual claims and vice versa. But, while undoubtedly true, this view has much scope for abuse along the lines of the no-true-Scotsman objection and, therefore, must be used advisedly when dealing with potential counterexamples. More importantly, Van Leeuwen does not really provide any evidence that the triplets of traits he identifies do regularly co-occur in the property space occupied by various propositional attitudes. He merely provides suggestions as to why some of the traits may co-occur. To show that a relatively clear distinction does exist—that factual beliefs and religious credences are clearly separable attractor positions in this space—it would be necessary to plot a representative sample of propositional attitudes in the property space and see whether they do cluster. What is more, such a study would need to be done cross-culturally, to avoid the possibility that the distinction is a WEIRD, post-secularization phenomenon (Henrich et al., 2010).

To better appreciate the distinctions among propositional attitudes, however, I would suggest that it would be better to consider the underlying reasons for any differences in how people think about mundane claims (I will use the term “mundane” as it does not beg the question that religious claims are not factual) as opposed to religious claims, and ideologies in general. These differences are systematic, maintained by social and cultural institutions and exist for a very good reason.

In evaluating claims made by others, people practice epistemic vigilance (Sperber et al., 2010). This vigilance can focus on the content of the claim (as per iii) as well as the source (as per c). Thus, when someone tells us where a petrol station is, we can consider whether the location they name is plausible as well as whether they are likely to know it. Which factors are considered depends as much on the social and epistemic context of the claim as its content. Scientific institutions such as blind review tend to promote content vigilance, religious ones such as the notion of the sacred promote source vigilance, while everyday strategies tend to use a mix. Why should this be the case? A belief tradition that eschews content vigilance may maintain beliefs independently of their truth since they will be judged to be plausible simply because they are believed by others. This is important in the case of religions. Unlike most mundane beliefs, and similarly to ideologies, the function of religious beliefs (and ideological beliefs in general) appears to be to promote prosocial attitudes, at least in the case of the so-called “big god” religions (Norenzayan et al., 2016). However, prosocial functionality is not tied to the truth of belief, unlike the functionality of beliefs that directly guide behavior (Talmont-Kaminski, 2013). You get the benefit of finding a store only if you had accurate directions. But the benefits (reputation, future cooperation, etc.) from helping someone else find a store are accrued even if what motivated you to do it was false (belief in a vengeful god, for example). So, the threat of supernatural punishment can work independently of the existence of the supernatural (Johnson and Krüger, 2004). But, for it to work, it must be believed in—in the sense that people have to consider the punishment a real possibility. Yet, since prosocial functionality is not tied to truth, many prosocial beliefs will be false. This means two things: prosocial beliefs have to be protected against potential counterevidence; and people who are epistemically vigilant have to be provided with reasons to accept the beliefs. This is achieved by promoting source vigilance over content vigilance in the case of prosocial beliefs.

The differences Van Leeuwen notes largely follow from this. Most obviously, focus on source vigilance makes prosocial beliefs, including religious beliefs, particularly vulnerable to authorities (c) and less so to evidence regarding the content (iii). Also, given their prosocial function, religious beliefs have a perceived normative orientation (a) and do not *have the function to directly* guide behavior (i). Finally, since logic is a major tool for content vigilance, drawing inferences (ii) is de-emphasized while, since the truth value of the claims is irrelevant to their function, elaboration upon them needn't be constrained (b).

Does this mean that Van Leeuwen is right? Yes and no. He is pointing to a significant distinction. However, I would argue that it is less straight-forward than he'd like to think. More importantly, putting it in terms of a distinction between types of propositional attitudes makes it sound purely psychological, and ignores underlying social and epistemological differences. The terminological question of beliefs vs. credences is secondary to identifying those causes. However, as already noted, religious claims have to be firmly believed in, in order to motivate prosocial behavior. So, “credences” cannot be too different from ‘beliefs’, a conclusion also reached by Boudry and Coyne (2016) on the basis of a different argument.

## Author contributions

The author confirms being the sole contributor of this work and approved it for publication.

### Conflict of interest statement

The author declares that the research was conducted in the absence of any commercial or financial relationships that could be construed as a potential conflict of interest.
